# Analysis of the effect of promoter type and skin pretreatment on antigen expression and antibody response after gene gun-based immunization

**DOI:** 10.1371/journal.pone.0197962

**Published:** 2018-06-01

**Authors:** Rajesh Vij, Zhonghua Lin, Kellen Schneider, Dhaya Seshasayee, James T. Koerber

**Affiliations:** Department of Antibody Engineering, Genentech, South San Francisco, California, United States of America; CEA Fontenay-aux-Roses, FRANCE

## Abstract

Monoclonal antibodies (mAbs) have enabled numerous basic research discoveries and therapeutic approaches for many protein classes. However, there still exist a number of target classes, such as multi-pass membrane proteins, for which antibody discovery is difficult, due in part to lack of high quality, recombinant protein. Here we describe the impact of several parameters on antigen expression and the development of mAbs against human claudin 4 (CLDN4), a potential multi-indication cancer target. Using gene gun-based DNA delivery and bioluminescence imaging, we optimize promoter type by comparing expression profiles of four robust *in vivo* promoters. In addition, we observe that most vectors rapidly lose expression, ultimately reaching almost background levels by three days post-delivery. Recognizing this limitation, we next explored skin pretreatment strategies as an orthogonal method to further boost the efficiency of mAb generation. We show that SDS pretreatment can boost antigen expression, but fails to significantly increase mAb discovery efficiency. In contrast, we find that sandpaper pretreatment yields 5-fold more FACS^+^ anti-CLDN4 hybridomas, without impacting antigen expression. Our findings coupled with other strategies to improve DNA immunizations should improve the success of mAb discovery against other challenging targets and enable the generation of critical research tools and therapeutic candidates.

## Introduction

Monoclonal antibodies (mAbs) bind their targets with high affinity and specificity, thus making them critical research tools and therapeutic agents. A wide variety of both *in vitro* selection technologies, such as phage or yeast display, and *in vivo* immunization methods exist for antibody discovery. For targets in which high quality, recombinant protein can be obtained, both avenues have proven to robustly deliver diverse panels of mAbs [1–3]. However, when recombinant protein is limiting, which is often the case for multi-spanning membrane proteins (MPs), existing antibody discovery strategies can fail to generate large panels of mAbs [4, 5]. Many MPs, including GPCRs and ion channels, have been shown to be dysregulated in diseases such as cancer, inflammation, diabetes, and even pain disorders and thus, not surprisingly, MPs comprise ~50% of known drug targets [6]. Despite this high therapeutic potential, there exist clinically approved mAbs against only two MP targets (CD20 and CCR4) [4, 7]. Strategies to increase the discovery efficiency of high quality mAbs will deliver larger panels for functional screening and ultimately, new therapeutic candidates against this challenging target class.

The ultimate goal for mAb discovery against MPs is to identify mAbs that selectively bind to the extracellular portion of MP when the MP is expressed in its native membrane environment and conformation. To enable efficient mAb discovery against MPs, a variety of different antigen formats have been explored. Since synthetic peptides are readily generated for any sequence, they typically provide a first pass antigen format. However, the peptides often do not accurately mimic the native conformation of the protein target and hence, fail to generate FACS^+^ antibodies. As such, antigen formats that reflect the native protein conformation are highly desirable. These formats can include whole cells, membrane fractions, or membrane-derived vesicles, which retain the protein in the native membrane environment [4, 8, 9]. However, the target of interest typically represents only a small fraction (<1–5%) of the total protein and thus, a large non-specific antibody response is often observed for these formats. Consequently, extensive counter-screening using multiple different cell lines is required, significantly expanding the cost and time for antibody discovery. DNA-based immunization using *in vivo* expression of the target cDNA provides another option [10]. In particular, DNA delivery represents an attractive strategy due to the ease of vector construction, low cost of gene synthesis, and *in vivo* expression of the native protein format [11]. However, the low and transient expression level and modest immune responses to DNA-based immunizations can limit the success of this strategy.

Optimization of both the expression vector and delivery method can improve the antibody response to DNA-based immunizations. On the plasmid side, the modular nature of the cDNA vectors enables changes in promoter [11–13], plasmid backbones [14], or genetic fusions to immune cell targeting moieties or immune stimulatory agents (*e*.*g*., T-cell epitopes or chemokines) [15–18]. On the delivery side, several physical, chemical, and viral delivery methods have been developed. Chemical (*e*.*g*., liposomes) and viral delivery can mediate efficient *in vivo* gene delivery, but few applications to mAb discovery have been described [19]. In contrast, physical delivery methods, such as biolistic, electroporation, or hydrodynamic tail vein (HTV), are routinely used for mAb discovery. HTV enables high level of expression in liver hepatocytes via tail vein delivery of large volumes of DNA and has enabled the mAb discovery against multi-spanning membrane proteins [11, 13]. However, extension to large species, such as rats and rabbits, is difficult and technical challenges with HTV injections can results in large variability between mice. Electroporation and biolistic delivery have proven to generate antigen-specific pAb responses in all species tested to date and require significantly less DNA than HTV. In contrast to HTV, these methods induce antigen expression in both keratinocytes and skin-resident dendritic cells such as dermal DCs or Langerhans cells, which can then drive robust immune responses [20, 21]. Here, we focus on gene gun-based delivery due to the relative ease of the approach, ability to work in a variety of species, and low DNA requirements. Gene guns enable biolistic gene delivery by using compressed gas to deliver DNA-coated gold particles into the skin [16, 22].

Many difficult to express proteins represent attractive diagnostic and therapeutic targets. Here we sought to develop a panel of mAbs against an emerging cancer target, human claudin 4 (CLDN4). CLDN4 is a 4-transmembrane protein that is a key component of tight junctions and regulator of paracellular permeability. Overexpression of CLDN4 occurs in a variety of epithelial and solid tumors, including pancreatic and ovarian cancers, and CLDN4 expression is also associated with more malignant phenotypes [23, 24]. Indeed early results with an anti-CLDN4 antibody were promising [25, 26]. However, antibody generation against CLDN4 is challenging in that recombinant protein is only obtainable via highly specialized methods, such as cell-free protein synthesis [27, 28]. Additionally, like most multi-spanning membrane proteins, CLDN4 possesses only a small extracellular footprint comprised of two short loops. These features make CDLN4 an attractive candidate for antibody discovery using DNA-based immunization.

While there are reports of the use of gene gun-based delivery to generate pAbs [16, 29], to our knowledge, no published efforts exist focused on how promoter type and skin treatment conditions impact both the kinetics of antigen expression as well as the efficiency of mAb discovery. Here we explore and optimize several key factors that enable efficient mAb generation using gene gun-based DNA delivery and apply these insights to discover novel CLDN4-specific mAbs. First, we use a luciferase-based *in vivo* imaging system to identify both pressures and promoters that mediate the highest expression levels after gene gun delivery. We observed that for most promoters the antigen expression level rapidly declines by ~10-fold each day post delivery. Using KO mice, we further demonstrate that in contrast to other delivery methods, this effect for gene gun delivery is not due to the production of TNFα or interferons [30–32]. Consequently, we sought to develop a different strategy beyond plasmid engineering to further improve gene gun-based immunizations. We reasoned that pretreating the skin with different agents, similar to those employed for topical immunizations of protein and DNA, could provide an additional stimulation to immune cells in the skin and thus enhance the antibody response to gene gun-based immunizations [33–37]. We demonstrate that pretreatment of the skin prior to gene gun delivery can increase antigen expression level as well as pAb titers against CLDN4. Finally, we demonstrate that skin pretreatment leads to a 5-fold increase in the number of FACS+ anti-CLDN4 mAbs isolated via hybridoma methods.

## Results

### Establishment of imaging system and effect of pressure

**Fig 1:**
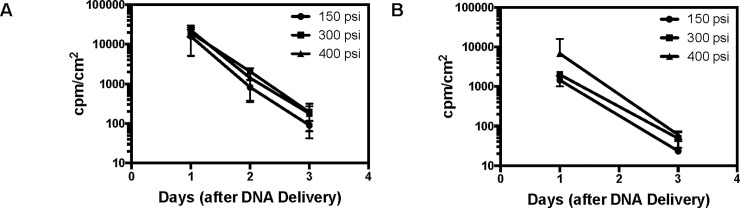
Luciferase expression profiles after gene gun-based DNA delivery. Luciferase expression profile in Balb/c (A) and C57BL6 (B) mice for three different gas pressures (150, 300, and 400 psi) at days 1, 2, and 3 post-delivery (n = 5 mice).

The level and kinetics of target expression can greatly impact the success of DNA-based immunization strategies [11, 13]. Furthermore, antigen expression during the first 2–3 days has shown to be critical to driving immune responses to gene gun-based immunizations [20, 21]. Recognizing this importance, we used an *in vivo* imaging system to monitor expression of a luciferase reporter gene after gene gun-based delivery. To minimize any effects of endotoxin, all DNA samples were prepared using a DNA purification kit designed to yield low endotoxin, in vivo grade DNA. We injected a plasmid (CAG-Luc) that encodes for luciferase under the control of the hybrid CMV/chicken beta-actin (CAG) promoter. We coated 0.3 μg of each plasmid onto 0.1 mg of gold microparticles and verified successful DNA capture using an agarose gel assay (data not shown). We then employed a gene gun device (Helios Gene Gun System) for *in vivo* delivery in two commonly used strains of mice (Balb/c and C57BL/6). We first evaluated the effect of delivery pressure (150, 300, and 400 psi) on both the initial level and kinetics of gene expression. At day one post-delivery, we observed no difference in luciferase expression among the different pressures (Fig 1A and 1B). Furthermore, while there was a trend towards slightly higher luciferase levels for the 300 and 400 psi groups at day three, it was not statistically significant. We therefore chose 400 psi for all future studies, since this is the commonly utilized pressure for skin delivery [38]. Interestingly, we observed a ~10-fold loss in luciferase expression each day, which resulted in almost a complete loss of expression after three days post-delivery.

### Effect of promoter on in vitro and in vivo expression

**Fig 2:**
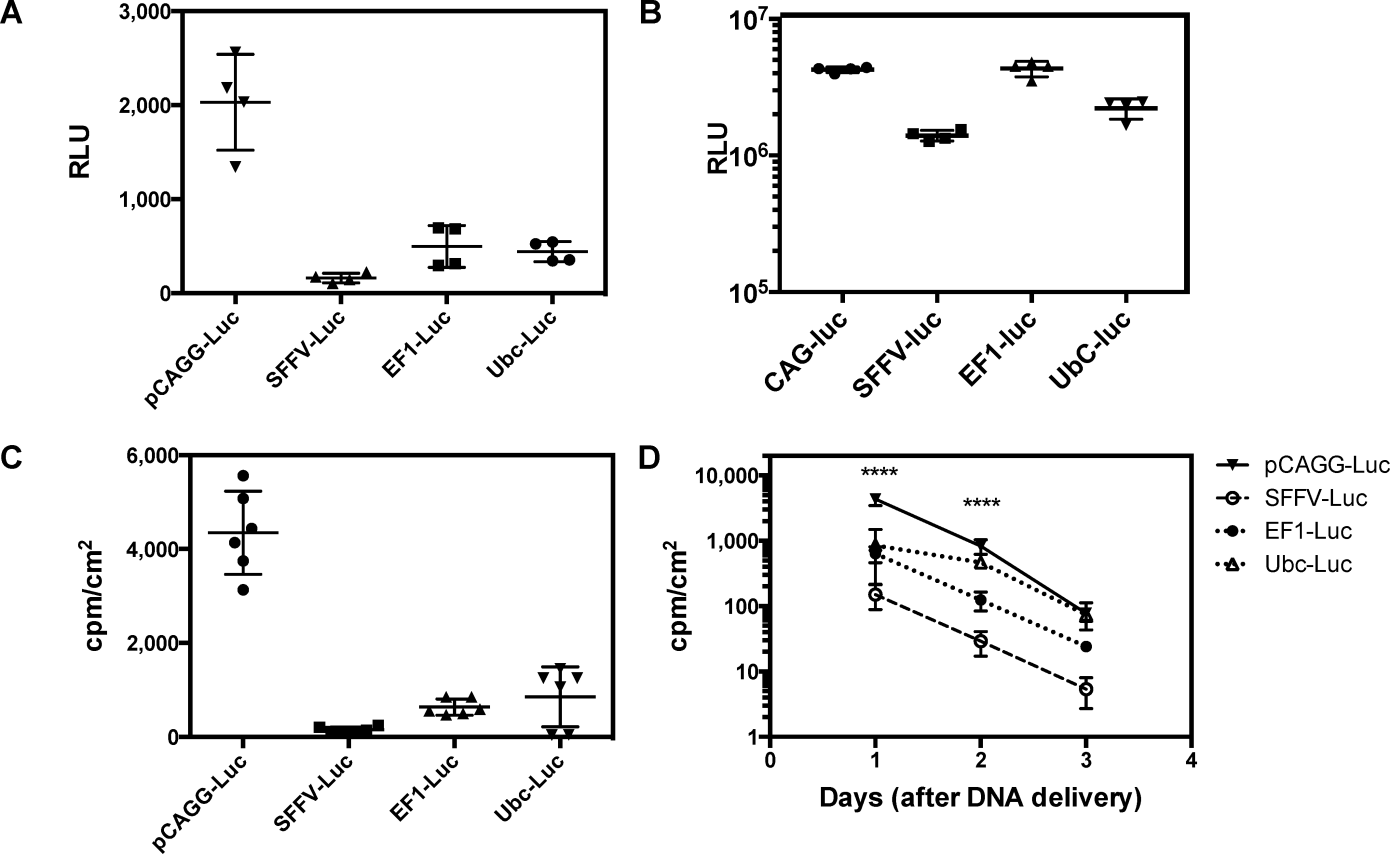
Comparison of *in vitro* and *in vivo* expression profiles of different promoters. (A) Luciferase expression levels in HaCat keratinocyte cells shows that CAG promoter greatly outperforms SFFV, EF1α, and UbC promoters (n = 4). (B) Luciferase expression levels in HEK293 cells similar expression from CAG and EF1α promoters (n = 4). (C) Luciferase expression levels in Balb/c mice shows that CAG promoter greatly outperforms SFFV, EF1α, and UbC promoters (n = 6; two shots in three mice). (D) Expression profile of each construct reveals a rapid decline in expression each day after delivery and ultimately, reaching background levels by day 3. Overall, the CAG promoter yields the highest expression at day 1 and day 2 (****: p<0.01, independent t-test).

Promoter strength contributes significantly to the antigen dose (both level and longevity of expression) and thus, the ultimate antibody response. Previous efforts to explore the role of different promoters on gene gun delivery efficiency have focused mainly on comparing the CMV promoter to either keratinocyte-specific (K14) or dendritic cell-specific promoters (CD11c or Fascin)[39]. In these cases, the CMV promoter yielded the highest expression level and antibody response. Therefore, we sought to compare the relative *in vivo* activities of a panel of robust promoters typically used for viral and non-viral gene delivery: CAG [11], human elongation factor 1 alpha (EF1a) [40], spleen focus forming virus (SFFV) [41], and ubiquitin C (UbC) [13]. We first evaluated the *in vitro* activities of these promoters after transient transfection into a human keratinocyte cell line (HaCaT) and a human embryonic kidney cell line (HEK293). Forty-eight hours post-transfection into HaCaT cells, the CAG-Luc construct yielded robust signal whereas only weak signal was observed for the other three constructs (Fig 2A). In contrast, all constructs expressed well in HEK293 cells (Fig 2B).

We sought to determine if expression in either of these cell lines was predictive of *in vivo* expression levels and thus, we next evaluated expression for each construct in Balb/c mice. At day one post-delivery, we again observed that the CAG-Luc yielded the highest level of luciferase expression (Fig 2C). Encouragingly, the correlation between *in vitro* activity in the HaCaT cells and *in vivo* activity in Balb/c mice was very strong, suggesting that expression in HaCaT cells could provide a relevant screening system for future constructs with enhanced activity. Overall, the kinetics of expression among the different promoters was similar, showing a 10-fold decrease in signal each day post-delivery (Fig 2D). This effect was slightly less pronounced for the UbC promoter, but the overall expression from this promoter was much less than expression from the CAG promoter.

### Effect of anti-viral response on in vivo expression levels

**Fig 3:**
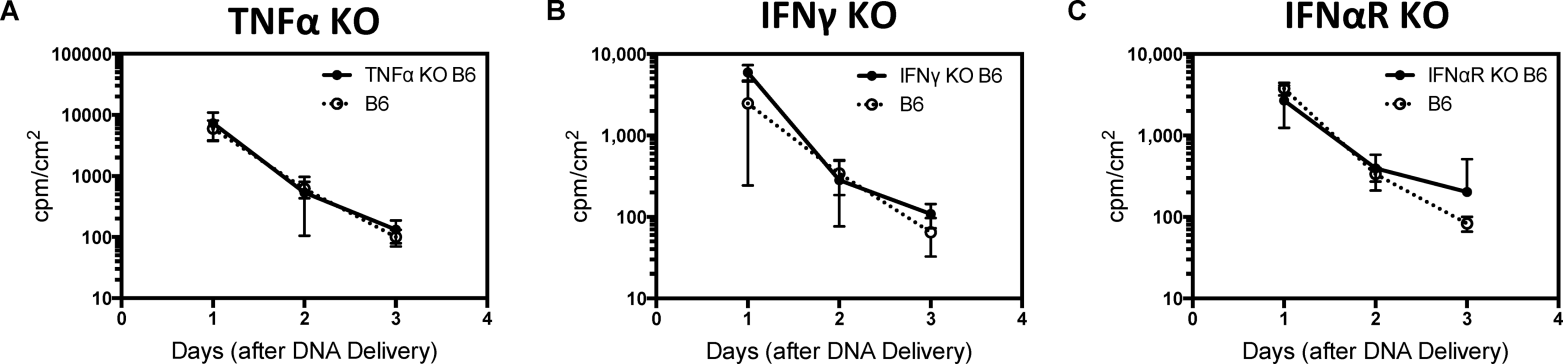
Comparison of luciferase expression profiles in TNFα, IFNγ, and IFNα receptor knockout (KO) mice. (A-C) A rapid decline in luciferase expression for the CAG promoter is observed in all three KO strains as well as the wild-type C57BL6 strain (n = 5).

The large decrease in luciferase expression levels after gene gun delivery is consistent with previous reports [42, 43]. Additionally, similar results have been observed after other methods of DNA delivery [30–32]. These studies demonstrated that an innate inflammatory response in the form of TNFα or interferon (IFNγ, IFNα, or IFNβ) production led to silencing of expression from viral promoters such as CMV [31, 32, 44]. Subsequent work has shown this is due to sensing of cytosolic DNA via a STING or Aim2-dependent pathway [45, 46]. Production of both TNFα and IFNγ has been observed after biolistic gene delivery. We thus considered the possibility that these cytokines may drive the loss in luciferase expression and potentially negatively impact antibody responses due to lower antigen expression [47]. To explore this scenario, we analyzed the initial level and kinetics of expression in TNFα, IFNγ, and IFNα receptor (IFNAR) KO mice compared to wild-type C57BL/6 mice. Surprisingly, we observed no statistically significant differences in either luciferase expression levels or kinetics (Fig 3). These results highlight that in contrast to other methods of DNA delivery, such as liposomes or viral delivery, the reduction in expression observed after gene gun delivery is likely not due to silencing via production of interferon and TNFα. Potentially, other mechanisms such as cell death may contribute to this reduction in expression[48]. Based upon our cumulative promoter studies, we chose CAG as the best candidate for future immunizations as it gave the highest overall expression level across the three day window.

### Effect of skin pre-treatment on expression and antibody response

**Fig 4:**
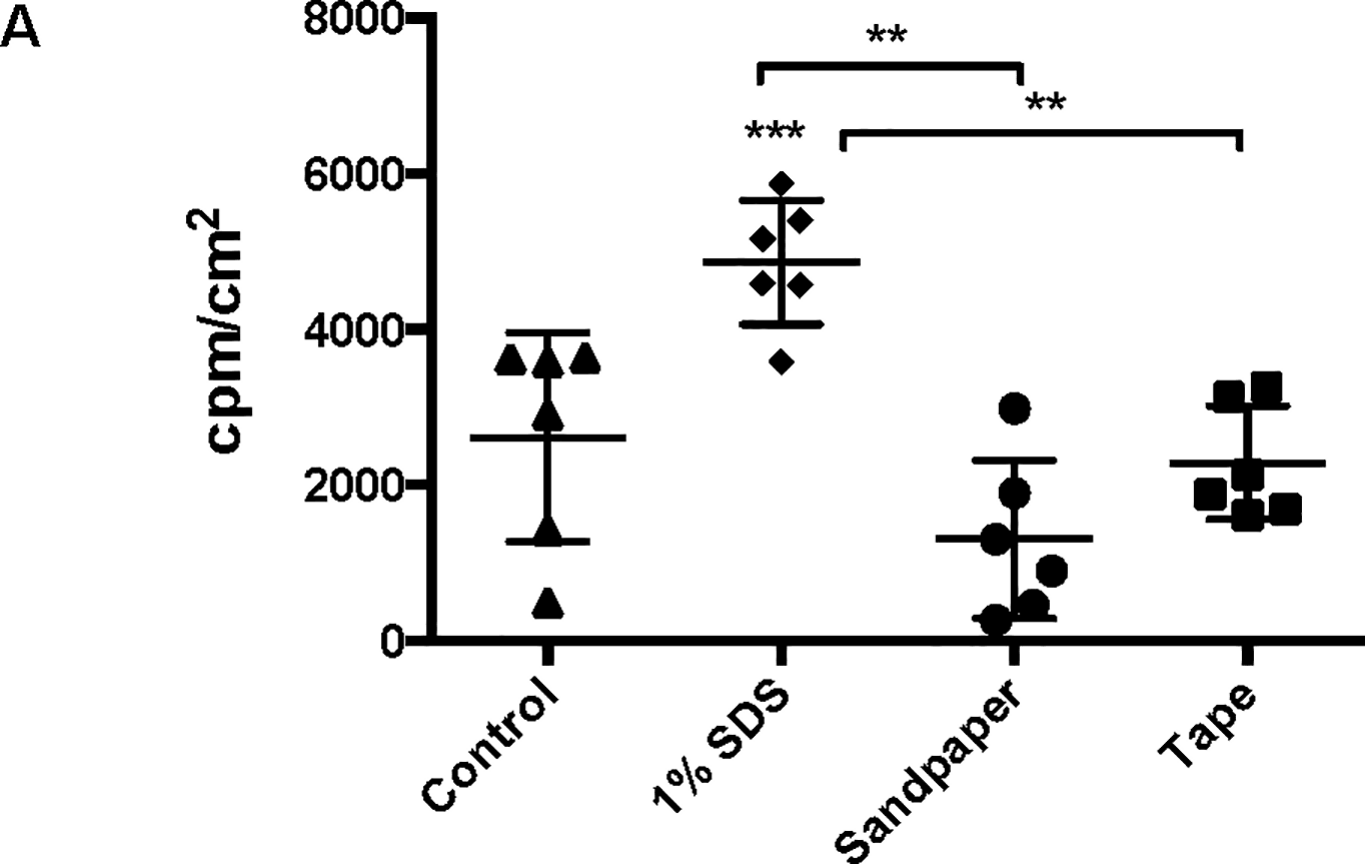
Effect of different skin pretreatment strategies on luciferase expression. (A) 1% SDS treatment leads to an increase in luciferase expression one day after delivery compared to other treatments and mock treated control (**, ***: p<0.01, independent t-test) (n = 6; two shots in three mice).

We next generated a CAG-huCLDN4 expression vector for subsequent DNA immunizations using the gene gun. Since the limited expression window might limit the effectiveness of additional plasmid engineering, we sought to establish other changes to the immunization protocol to boost the efficiency. The stratum corneum in the skin provides a significant barrier for biolistic gene delivery to the skin and likely reduces DNA transduction of the underlying epidermis and dendritic cells (Langerhans and dermal). We reasoned that pretreating the skin with an agent designed to disrupt the stratum corneum could improve expression in the underlying cell types and/or provide an immune stimulus to activate skin-resident immune cells as observed for previous topical DNA delivery methods [33, 34]. We first evaluated the effect of three different skin pretreatments (SDS, sandpaper, and tape stripping) on expression level. Similar treatments have previously been shown to improve antibody response after protein delivery to the skin [35–37]. Animals were treated first with Nair to remove hair and then treated as described in the Materials and Methods prior to gene gun delivery of CAG-Luc. We then monitored luciferase expression level one day later. Interestingly, only pretreatment with 1% SDS led to a significant increase in luciferase expression compared to the other two pretreatments and an untreated control (Fig 4A).

**Fig 5:**
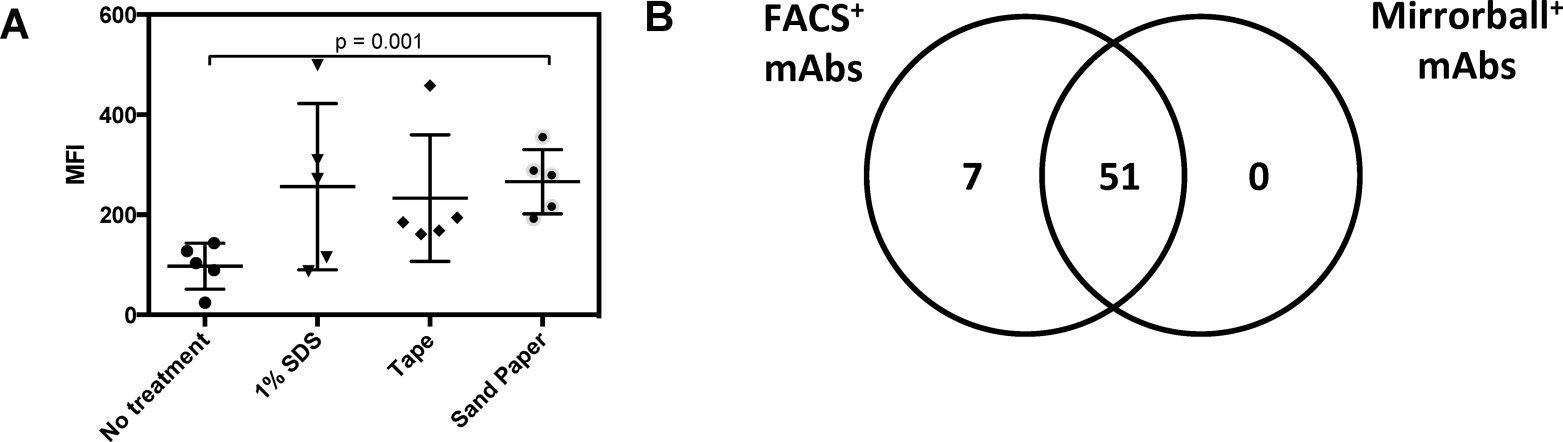
Effect of different skin pretreatment strategies on anti-huCLDN4 Ab response. (A) Sandpaper treatment results in a significant increase in MFI against HEK293 expressing huCLDN4 compared to other treatments and mock treated control (n = 5; p < 0.001, independent t-test). Samples were analyzed at a 1:100 dilution. (B) A comparison of FACS- and Mirrorball-based screen of hybridoma supernatants on HEK293 huCLDN4 and parental HEK293 cells demonstrates an 89% (51/57) overlap between the two methods.

We next asked how these different pretreatments would impact the antibody response to CLDN4. To analyze this effect, we used FACS to detect the ability of the polyclonal Abs (pAb) to recognize the native conformation of CLDN4. We used mean fluorescence intensity (MFI) values as a metric to compare the levels of antibody-CLDN4 binding between difference sera samples. An increase in MFI would represent a higher concentration of antibody capable of binding CLDN4. After four weeks of gene gun delivery, the sand paper pretreatment group had a statistically significant increase in MFI compared to the negative control (p<0.001, Fig 5A). While the 1% SDS and tape pretreatment groups had a trend towards higher MFIs, they were not statistically significant (p = 0.073 and p = 0.053, respectively). Interestingly, since only the 1% SDS group showed improved gene expression, the elevated antibody response for the sandpaper group is likely due to other mechanisms such as enhanced immune cell activation [49–51].

### Effect of skin pretreatment on generation of CLDN4-specific mAbs

As our ultimate goal was to generate a large panel of anti-CLDN4 mAbs, we next generated hybridomas from mice in three different groups (1% SDS, sand paper, and no treatment). Each animal group was subjected to five weekly doses of three shots. While FACS-based screening is the gold standard to identify antigen-specific mAbs, it can be labor-intensive and require large numbers of cells. As such, we sought to compare FACS-based screening results with screening results from a homogeneous, no-wash cell-based assay performed using a Mirrorball plate reader [52]. In this assay, antibody samples, the corresponding secondary antibody, and cells are mixed and allowed to bind for two hours. The Mirrorball plate reader then uses laser excitation and selective detection of fluorescence emission from cells that settled to the bottom of the plates. Individual cells are detected via labeling of the nucleus with DRAQ5. The fluorescent signal corresponding to the amount of secondary detection antibody bound is then calculated by subtracting background fluorescence from unbound antibody in the solution. We screened supernatants from all 318 IgG^+^ hybridomas with both assays. Overall, FACS-based screening of hybridoma supernatants on a CLDN4 HEK293 stable cell line identified 58 total CLDN4-specific mAbs whereas Mirrorball screening identified 51 total CLDN4-specific mAbs. Overall, the correlation was quite good with ~88% (51/58) of CLDN4-specific mAbs positive in both assays (Fig 5B). The seven hybridomas that were only FACS+ exhibited very low MFIs (10–30) and thus, potentially differences in assay format (*e*.*g*., wash vs. no wash or laser intensity) could account for these results.

Comparing the mAb screening results among the three pretreatment groups revealed some interesting trends (Table 1). The 1% SDS treated group yield only slightly higher number of IgG+ hybridomas with a similar percentage of CLDN4-specific clones (15.3% vs. 12.7%), suggesting that the higher expression we observed for this group failed to translate into more CLDN4-specific mAbs. In contrast, the sandpaper treated group yield ~3.2-fold more IgG+ hybridomas and had a higher percentage of CLDN4-specific mAbs compared to the untreated control (21.4% vs. 12.7%). Collectively, our results highlight that combining a CAG expression vector with sandpaper pretreatment can yield a large panel of target-specific mAbs against a multi-pass membrane protein.

**Table 1:** Antibody discovery efficiency using different pretreatments.

Pretreatment	IgG+ hybridomas	FACS+ hybridomas	% FACS+
None	55	7	12.7
1% SDS	85	13	15.3
Sand paper	178	38	21.4

## Discussion

MAbs provide essential research tools and therapeutic agents for a wide range of proteins. However, the generation of large panels of mAbs against difficult to express proteins, such as disulfide-rich domains or multi-pass MPs, has proven challenging. Many alternatives to recombinant protein have been explored as antigens for these types of targets. DNA-based immunizations, in particular, are attractive due to the ease of generation and modularity of DNA expression vectors. In the case of gene gun-based delivery, much of the optimization was done prior to the discovery of newer vector designs and other topical immunization strategies [16, 17, 39]. Furthermore, much of the previous optimization of DNA-based immunizations has focused on pAb titers as the final readout and not on how these parameters impact the final mAb discovery efficiency.

Focusing first on promoter type, we have found that *in vitro* expression in the HaCat human keratinocyte cell line correlates well with *in vivo* expression levels (Fig 2A vs. Fig 2C). This finding will enable additional efforts to further improve expression levels. For example, while the CAG promoter provides the highest initial expression level, the UbC promoter exhibits more stable expression during the first 48 hours and thus, a hybrid CAG-UbC promoter that combines both of these features would be ideal (Fig 2D). The full UbC promoter encompasses several elements derived from the human UbC gene: a promoter, several enhancers, the first exon and first introns of UbC [53]. A CAG-UbC hybrid could be constructed by replacing promoter element from chicken beta-actin in CAG with the corresponding promoter element from UbC. Alternatively, the enhancer or intron regions could be swapped. In this case, the HaCat cell line could be used to screen a panel of CAG-UbC hybrid promoters for those promoters that retain high level of expression prior to final *in vivo* testing to determine the duration of expression. Another emerging alternative is the design of synthetic promoters using parts from endogenous promoters [54–56]. Here, libraries of endogenous or completely synthetic enhancers, transcription factor binding sites, and other regulatory elements are assembled and subsequently screened in a cell-type of interest. In many cases, these synthetic promoters can achieve expression levels equal to or better than the benchmark CMV promoter and these new promoters should avoid many of the downsides of virus-derived promoters, such as silencing [30–32].

Regardless of promoter type, the antigen expression rapidly decreases to background within three days after gene gun-mediated delivery. Part of this effect may due to death of antigen-expressing keratinocytes as significant levels of dead cells have been detected after gene gun delivery[48]. Previous reports indicate expression during this initial window is critical to achieve high levels of antigen-bearing DCs and thus, initiate an immune response [20, 21]. As such, the identification of strategies, beyond antigen vector optimization, to boost and prolong expression may further improve the final antibody response. For example, co-delivery of an anti-apoptotic factor, such as Bcl-xL or XIAP, has been shown to significantly boost humoral and cell-mediate immune responses [57, 58]. While this treatment has been shown to promote survival of antigen-bearing DCs, it may also promote survival of antigen-expressing keratinocytes. Somewhat surprisingly, enhanced immune responses have also been observed upon co-delivery of low doses of activity- pro-apoptotic caspase 2 or caspase 3[43]. Future studies aimed at comparing both of these strategies in the context of a multi-pass MP will be particularly interesting as presumably some of the MP must be released from dying cells to prime immune cells.

As an orthogonal strategy to boost gene gun-mediated gene delivery, we explored several permeation/abrasion treatments prior to delivery. Previous reports indicate that disruption of the stratum corneum leads to the production of proinflammatory cytokines (*e*.*g*., TNFα, IL-1α, IL-1β), T_H_2 promoting cytokines (*e*.*g*., IL-4, IL-10, and TSLP), and GM-CSF [50, 51]. One direct consequence of this effect is the activation of skin-resident Langerhans cells (and likely dermal cells) that results in upregulation of MHC class II and other co-stimulatory molecules [50]. In our case, we hypothesized that these same mechanisms that have been observed for protein-based immunizations would contribute to improving the gene gun-based immunizations. While the 1% SDS pretreatment led to an increase in antigen expression, this strategy failed to significantly increase the number of FACS^+^ CLDN4 mAbs. Quite strikingly, the sandpaper pretreatment led to a marked improvement in the number of FACS^+^ CLDN4-specific mAbs generated, likely without significantly improving antigen expression level, suggesting pretreatment methods can provide an enhancement effect independent of antigen expression. As such, we envision that future efforts to combine modified pretreatment strategies with increased antigen expression methods will lead to additional improvements in antibody discovery efficiency using DNA-based immunizations.

Difficult to express proteins, such as multi-pass MPs, comprise a largely untapped class of therapeutic and diagnostic targets for mAbs. Here we have explored how parameters such as promoter and skin pretreatment impact both antigen expression and ultimate discovery efficiency of FACS^+^ mAbs against CLDN4. These mAbs will enable further validation of huCLDN4 as a potential cancer target. Ultimately, applying our findings with other strategies to boost DNA-based immunizations for other difficult to express targets will advance mAb discovery to uncover new biological insights and enable therapeutic targeting of these challenging proteins.

## Materials and methods

### Vector construction

We modified a previously described vector to express the cDNA encoding for the target antigen (luciferase or human CLDN4) under control of a CAG promoter, which is comprised of the CMV early enhancer, chicken beta-actin promoter, and rabbit beta-globin splice acceptor [11]. This cassette also contained a woodchuck Hepatitis virus post-transcriptional regulatory element (WPRE) to help stabilize mRNA. To generate the luciferase vectors with various promoters, we replaced the CAG promoter with sequences for human elongation factor 1 alpha (EF1α) [13], spleen focus forming virus (SFFV) [41], and ubiquitin C (UbC) [40]. All vectors were constructed using gene synthesis (Genewiz). All DNA samples for *in vivo* studies were purified using EndoFree Plasmid kits (Qiagen, Cat. 12362) to avoid endotoxin contamination.

### In vitro bioluminescence assay

HEK293 cells (ATCC, Cat. #CRL-1573) and HaCat cells (Addexbio, Cat. #T0020001) were cultured in DMEM with 10% FBS. Both cell types were plated in 96-well plates and transiently transfected with each Luc vector using TransIT-X2 (Mirus, Cat. MIR6000) according to the manufacturer’s instructions. Approximately forty-eight hours post-transfection, samples were processed using the Dual-Luciferase Reporter Assay (Promega, Cat. #E1910). Briefly, 20 μL of 1x Passive Lysis Buffer were added to each well and the plates were shaken at 25°C for 15 min. Then, 20 μL of cell lysate was mixed with 100 μL of LAR II reagent and resulting signal was read on an Envision plate reader (Perkin Elmer). Measurements were performed in quadruplicate.

### Bioluminescence imaging

Animals are anesthetized using isoflurane and injected intra-peritoneally with 100 μl of Luciferin D (Molecular Probes; Cat. #L2912) at 150 mg/kg and placed in imaging chamber. During image acquisition animals were maintained on anesthesia using a nose cone delivery system, and body temperature was regulated using a thermostatically controlled warm pad. Bioluminescence imaging was achieved using a CCD camera capturing light in the range of 500–700 nm. Data was acquired for 5 min and the data analyzed is for 100-second time interval. Animals were imaged for luciferase expression at different time points.

### Animal immunizations

All the studies were conducted in mice (6–12 weeks old). BALB/c and C57BL6 mice were obtained from Charles River Laboratories. The IFNAR knockout C57BL6 mice from Genentech Inc., and the TNFα knockout and IFNγ knockout C57BL6 mice were obtained from Jackson Laboratory. Approval of the study design was obtained from the Genentech Institutional Animal Care and Use Committee prior to the start of this work.

### Gene gun methods and hybridoma generation

DNA/gold particle bullets are prepared using previously published methodologies with a few refinements [22, 29]. Each bullet for DNA was prepared to contain a total of 0.3 μg of DNA coated onto 0.1 mg of gold particles (BioRad, Cat. #1652264). Bullets were stored at 4°C in the dark in the presence of desiccant pellets.

All the procedures were conducted under anesthesia. The abdominal and thoracic areas were shaved with a clipper or a depilatory cream was applied (Nair). The cream was left on for 2 min, wiped off, cleaned with sterile water and dried. At this point mice are ready for DNA delivery. For initial pressure and promoter studies, five mice were immunized with a single shot from the gene gun (BioRad, Cat. #1652431) at a helium pressure of 150, 300, or 400 psi. For the KO mice study, five mice were injected with a single shot. For CLDN4 immunizations, five mice were each injected with three shots (1 shot at thoracic and 2 shots at abdominal region) at weekly intervals for a total of 5 weeks.

For the pretreatment study, skin was treated as described below with different reagents prior to DNA delivery using the gene gun. 1) Sodium dodecyl sulfate (SDS): 1% SDS or PBS was applied to the depilated skin for 5 min (wiped 10x) and then wiped with water to remove SDS from the skin 2) Sand paper: fine grade sandpaper (3M; Cat. #40320SBP-UF4, 320 fine, gas sterilized) was applied to the skin under slight pressure and rubbed 5 times in same direction and 3) Tape: Scotch tape was applied to the skin and removed for a total of 10 repeats. Hybridomas were generated from each group using the same initial number of cells for the fusion and further processed as previously described [11].

### Flow cytometry screening of antibodies

To monitor the ability of the antibodies to the extracellular portion of CLDN4 on cells, 300,000 cells (either control HEK293 cells or HEK293 cells stably expressing huCLDN4) were incubated with 100 μL of sample in FACS buffer (PBS + 2% FBS) on ice for 40 min. For the polyclonal Abs, we incubated cells with 100 uL of a 1:100 dilution of mouse sera. For the monoclonal Abs, we incubated cells with 100 uL of hybridoma supernatant. The cells were washed and goat anti-mouse PE secondary antibody (Jackson Immunoresearch; Cat. #115-116-071), diluted at 1:1000, was added to each well and incubated for 30 min at 4°C. The cells were washed twice with FACS buffer. The data was acquired on FACS Caliber (BD Bioscience, USA) and analyzed by Flowjo software (Flowjo LLC, USA). Forward and side scatter parameters were used to eliminate debris and dead cells from the analysis. The mean fluorescence intensity (MFI) values for PE were measured to detect to the binding of the CLDN4 antibody bound to the extracellular portion of CLDN4.

### Mirrorball-based antibody screening

Both 293 hCLD4 and 293 control cells were washed in DMEM media and resuspended at 100,000 cells per mL. 1 mM DRAQ5 (ThermoFisher; Cat. #62251) and 0.8 mg/ml of anti-mouse IgG-Alexa 488 (Jackson Immunoresearch; Cat. #115-546-062) were added to the each cell suspension. 20 μL of the resultant cell suspension was added to each well followed by addition of 10 μL of hybridoma supernatant. The plate was sealed and incubated at 25°C for two hrs. After two hrs, the plate was read with a Mirroball (TTP Labtech Ltd, UK). The results were exported as fluorescence intensity after subtracting the background fluorescence from unbound antibody in solution.
